# Doxycycline Restores Gemcitabine Sensitivity in Preclinical Models of Multidrug-Resistant Intrahepatic Cholangiocarcinoma

**DOI:** 10.3390/cancers17010132

**Published:** 2025-01-03

**Authors:** Annamaria Massa, Francesca Vita, Caterina Peraldo-Neia, Chiara Varamo, Marco Basiricò, Chiara Raggi, Paola Bernabei, Jessica Erriquez, Francesco Leone, Massimo Aglietta, Giuliana Cavalloni, Serena Marchiò

**Affiliations:** 1 Candiolo Cancer Institute, FPO-IRCCS, 10060 Candiolo, Italy; annamaria.massa@ircc.it (A.M.); fr.vita@outlook.it (F.V.); varamo.chiara@gmail.com (C.V.); paola.bernabei@ircc.it (P.B.); jessica.erriquez@ircc.it (J.E.); massimo.aglietta@ircc.it (M.A.); giuliana.cavalloni@ircc.it (G.C.); 2 Department of Oncology, University of Torino, 10060 Candiolo, Italy; marco.basirico@unito.it; 3 Laboratory of Cancer Genomics, Fondazione Edo ed Elvo Tempia, 13900 Biella, Italy; caterina.peraldoneia@fondazionetempia.org; 4 Department of Experimental and Clinical Medicine, University of Firenze, 50134 Firenze, Italy; chiara.raggi@unifi.it; 5 Department of Oncology, ASL BI, Nuovo Ospedale degli Infermi, 13875 Ponderano, Italy; francesco.leone@aslbi.piemonte.it

**Keywords:** doxycycline, drug repurposing, cancer therapy

## Abstract

Background/Objectives: Intrahepatic cholangiocarcinoma (iCCA) is a malignant liver tumor with a rising global incidence and poor prognosis, largely due to late-stage diagnosis and limited effective treatment options. Standard chemotherapy regimens, including cisplatin and gemcitabine, often fail because of the development of multidrug resistance (MDR), leaving patients with few alternative therapies. Doxycycline, a tetracycline antibiotic, has demonstrated antitumor effects across various cancers, influencing cancer cell viability, apoptosis, and stemness. Based on these properties, we investigated the potential of doxycycline to overcome gemcitabine resistance in iCCA. Methods: We evaluated the efficacy of doxycycline in two MDR iCCA cell lines, MT-CHC01R1.5 and 82.3, assessing cell cycle perturbation, apoptosis induction, and stem cell compartment impairment. We assessed the in vivo efficacy of combining doxycycline and gemcitabine in mouse xenograft models. Results: Treatment with doxycycline in both cell lines resulted in a significant reduction in cell viability (IC_50_ ~15 µg/mL) and induction of apoptosis. Doxycycline also diminished the cancer stem cell population, as indicated by reduced cholangiosphere formation. In vivo studies showed that while neither doxycycline nor gemcitabine alone significantly reduced tumor growth, their combination led to marked decreases in tumor volume and weight at the study endpoint. Additionally, metabolic analysis revealed that doxycycline reduced glucose uptake in tumors, both as a monotherapy and more effectively in combination with gemcitabine. Conclusions: These findings suggest that doxycycline, especially in combination with gemcitabine, can restore chemotherapy sensitivity in MDR iCCA, providing a promising new strategy for improving outcomes in this challenging disease.

## Simple Summary

Intrahepatic cholangiocarcinoma (iCCA) is a rare but aggressive liver cancer with limited treatment options, especially when tumors become resistant to standard drugs like gemcitabine. This study investigates whether doxycycline, an antibiotic, can restore the effectiveness of gemcitabine in treating drug-resistant iCCA. Using in vitro models, we found that doxycycline reduces the survival and stem-like properties of cancer cells. In mouse models, the combination of doxycycline and gemcitabine significantly shrank tumors that were otherwise resistant to treatment. These findings suggest that doxycycline could be repurposed to improve outcomes for patients with this challenging cancer, offering a potential new combination therapy for clinical use.

## 1. Introduction

Intrahepatic cholangiocarcinoma (iCCA) is a malignant neoplasm originating from the biliary epithelium within the liver. Although relatively rare in Western countries, its incidence has been steadily increasing worldwide in recent years [1,2]. Due to late diagnosis, approximately half of patients present with inoperable tumors, leading to rapid progression to an advanced or metastatic stage. Consequently, the prognosis for these patients is bleak, with an overall survival of less than 6 months and a 5-year survival rate below 10% [3,4]. The first-line standard-of-care for advanced disease remains chemotherapy, mainly based on gemcitabine + cisplatin (GemCis) [5]. Recently, the phase III trial TOPAZ-1 demonstrated that adding the anti-PD-L1 antibody durvalumab to GemCis improves overall survival (OS), progression-free survival (PFS), and response rates (RR) [6,7]. Consequently, the FDA and EMA have approved this combination as the standard of care for unresectable, metastatic, and untreated biliary cancers [8]. Upon progression, second-line treatment with 5-fluorouracil (5-FU) and oxaliplatin (FOLFOX) is typically recommended [9]. However, while these regimens are aggressive, they often prove ineffective against iCCA due to the development of chemotherapy-induced cross-resistance to multiple drugs.

Doxycycline, an antibiotic derivative of tetracycline, exhibits antitumor effects [10]. In vitro studies have demonstrated its ability to inhibit adhesion, migration, invasion, and proliferation of both cancer cells and cancer stem cells. These effects are accompanied by induction of apoptosis, dysregulation of metabolic pathways, alteration of mitochondrial protein synthesis, autophagy, epithelial-to-mesenchymal transition, and activation of inflammatory pathways. In vivo, doxycycline treatment results in reduced numbers of cancer stem cells, impaired angiogenesis, delayed growth of primary tumors, and reduced metastatic dissemination [11,12,13]. These findings have been corroborated in preclinical models of various cancers including colorectal cancer [14,15], melanoma [16,17], pancreatic cancer [13,18], oral squamous-cell carcinoma [19], hepatocellular carcinoma [20], cervical cancer [21,22], breast cancer [23,24], and prostate cancer [25]. Clinical studies have also hinted at the impact of doxycycline on the cancer stem cell compartment; pre-operative administration of the drug to patients with breast cancer led to reduced expression of the stemness markers CD44 and ALDH1 in tumors compared with matched pre-treatment biopsies [26].

We have generated and characterized patient-derived cell models of iCCA exhibiting primary or acquired resistance to gemcitabine, rendering them refractory to additional chemotherapeutics including 5-FU [27]. These cell lines serve as ideal models for validating new druggable targets and pathways to potentially overcome multidrug resistance. In a recent study, we identified doxycycline as a potential therapeutic option for CCA [28]. Here, we characterize the in vitro and in vivo efficacy of doxycycline-based treatments in our multidrug-resistant iCCA models, to support a future introduction of this drug in first-line therapeutic regimens.

## 2. Materials and Methods

### 2.1. Cell Lines and Drugs

The MT-CHC01R1.5 (RRID:CVCL_C1L3) and 82.3 (RRID:CVCL_A7NJ) multidrug-resistant iCCA cell lines, exhibiting acquired and primary resistance to gemcitabine, respectively, were isolated and characterized at our institution [27,28,29]. Both cell lines were cultured in KnockOut-DMEM/F-12 medium (ThermoFisher Scientific, Waltham, MA, USA), supplemented with 10% fetal bovine serum (FBS), Hepes buffer, 100 U/mL penicillin, and 100 μg/mL streptomycin (P/S) (all from Sigma-Aldrich, St. Louis, MO, USA). Gemcitabine hydrochloride (SANDOZ, Novartis, Siena, Italy) and doxycycline (Merk Millipore, Milan, Italy) were dissolved in water for injection and stored as aliquots in working solutions.

### 2.2. Cell Viability Assay

Cells (2500 per well of 96-well plates) were seeded under optimal culture conditions. The following day, escalating doses of doxycycline (0.625–1.25–2.5–5–10–20 μM) were added and incubated for 72 h. Cell viability was assessed using the Cell Titer-Glo kit assay (Promega, Milan, Italy) following the manufacturer’s protocol, and luminescence was measured with a Glomax microplate reader (Glomax-Multi Detection System, Promega; RRID:SCR_018614). The half maximal inhibitory concentration (IC_50_) was determined using Calcusyn software v.2.0 (Biosoft, Cambridge, UK; RRID:SCR_020251) based on the Chou–Talalay method.

### 2.3. Flow Cytometry Analysis of Cell Cycle and Apoptosis

To evaluate the cell cycle, 2 × 10^6^ cells were seeded into 10 cm dishes and allowed to attach overnight. The following day, cells were treated with doxycycline (15 µg/mL) or gemcitabine (1.5 µM) for 72 h. After treatment, cells were detached, fixed in ice-cold 70% ethanol, and incubated in a solution containing 10 μg/mL Propidium Iodide (PI), 0.1% Triton X-100, and 100 μg/mL RNase (ThermoFisher Scientific) in PBS at 37 °C for 15 min in the dark. To assess apoptosis, 1 × 10^5^ cells were seeded per well in 6-well plates with optimal culture medium and allowed to attach overnight. Cells were then treated as described above. After treatment, cells were incubated in binding buffer (Bender MedSystems, Wien, Austria) for 30 min at 4 °C in the dark. They were subsequently resuspended in binding buffer containing Annexin V-APC (Bender MedSystems) and PI (50 µg/mL; ThermoFisher Scientific) and incubated for 1 h at 4 °C in the dark. For both assays, samples (3 × 10^4^ cells per experimental point) were acquired using a CyAn ADP™ flow cytometer (Beckman Coulter, Brea, CA, USA; RRID:SCR_019666), and the data were processed using FlowJo™ software v.7.6.3 (BD Biosciences, Franklin Lakes, NJ, USA; RRID:SCR_008520).

### 2.4. Cholangiosphere Formation Assay

Cells (2.5 × 10^5^ per well) were seeded into 24-well ultra-low attachment plates and cultured in stem cell serum-free medium consisting of DMEM-F12, 0.4% BSA, 4 μg/mL insulin (all from Sigma), 1× B27 Supplement (ThermoFisher Scientific), 200 ng/mL human EGF (PeproTech, London, UK), and P/S. After 7 days of treatment with gemcitabine (1.5 µM) or doxycycline (15 µg/mL), cholangiospheres with diameters exceeding 50 µm were quantified under an optical microscope (DMIL, Leica Microsystems GmbH, Wetzlar, Germany).

### 2.5. In Vivo Experiments

Non-Obese Diabetic-Severe Combined Immunodeficient (NOD/SCID; RRID:IMSR_CRL:394) female mice aged 4–6 weeks (Charles River Laboratory, Wilmington, MA, USA) were housed under sterile conditions in micro-isolator cages at the animal facilities of Candiolo Cancer Institute, FPO-IRCCS. MT-CHC01R1.5 cells (3.0 × 10^6^) were suspended in growth factor-reduced basement membrane matrix (Matrigel, BD Biosciences) at a final concentration of 50% *v/v* and injected subcutaneously into the right flank. Three weeks post-injection, when tumors reached a volume of approximately 50–100 mm^3^, mice (6 per experimental group) were randomly assigned to receive one of the following treatments: drug vehicle, gemcitabine (25 mg/kg), doxycycline (20 mg/kg), or their combination. Treatments were administered intravenously twice a week. Tumor growth was monitored using calipers, and volumes were calculated with the formula V = (A×B^2^)/2 (where V = tumor volume, A = largest diameter, B = smallest diameter). At the end of the experiment, tumors were excised and weighed.

Glucose uptake was visualized in live animals using an IVIS Lumina II instrument and ex vivo with an IVIS Spectrum CT instrument (PerkinElmer, Waltham, MA, USA) following tail vein injection of XenoLight RediJect 2-DeoxyGlucose-750 (Caliper Life Sciences, Waltham, MA, USA). Image analysis was conducted with Living Image software v.4.8.2 (PerkinElmer, Caliper Life Sciences; RRID:SCR_014247). Glucose uptake by tumor xenografts was quantified by calculating the ratio of total fluorescence emitted to the total number of tumor mass voxels.

### 2.6. Statistical Analysis

All statistical analyses were conducted with GraphPad Prism v.10 (RRID:SCR_002798). Two-way ANOVA was used to analyze cell and tumor growth, as well as the differences in terms of drug response. One-way ANOVA (multiple comparisons) was used to compare the different experimental points.

## 3. Results

### 3.1. Doxycycline Reduces Cell Viability and Causes Apoptosis in Multidrug-Resistant iCCA Cell Lines

**Figure 1:**
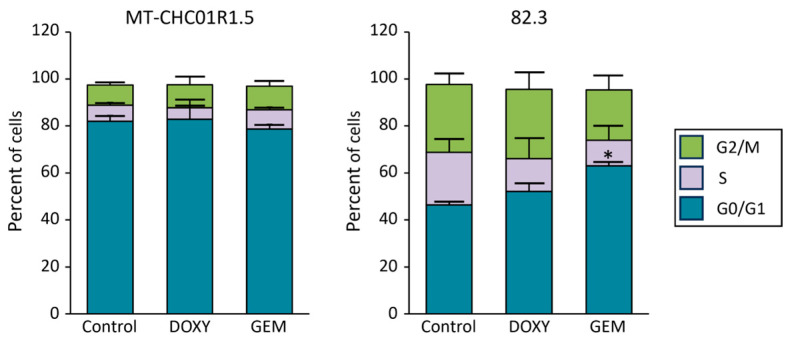
Cell cycle analysis. MT-CHC01R1.5 and 82.3 cells were treated with doxycycline (15 µg/mL) or gemcitabine (1.5 µM) for 48 h, and cell cycle distribution was analyzed using PI staining and flow cytometry. The bars show the mean ± SEM percentage of cells in each phase of the cell cycle from three independent experiments. Control: no treatment; DOXY: doxycycline; GEM: gemcitabine. * *p* < 0.05 for GEM vs. Control (two-way ANOVA).

We initially evaluated the effect of doxycycline on the viability of two multidrug-resistant iCCA cell lines: MT-CHC01R1.5 (with acquired resistance) and 82.3 (with primary resistance) [27,28,29]. We tested different concentrations of doxycycline, observing reduced viability of both cell lines, with IC_50_ values of 14.85 ± 1.8 µg/mL for MT-CHC01R1.5 and 15.97 ± 3.38 µg/mL for 82.3 cells. To determine if this reduction in viability was linked to cell cycle disruption, we conducted PI-based flow cytometry analysis. The analysis showed no significant changes in cell cycle distribution for either MT-CHC01R1.5 or 82.3 cells treated with doxycycline compared to the control. For comparison, gemcitabine was also included in the cell cycle studies, as the investigated cell lines are resistant to this drug. As expected [27,28], gemcitabine did not affect the cell cycle distribution of MT-CHC01R1.5 cells and produced only a slight increase in the percentage of 82.3 cells in the G0/G1 phase (Figure 1).

**Figure 2:**
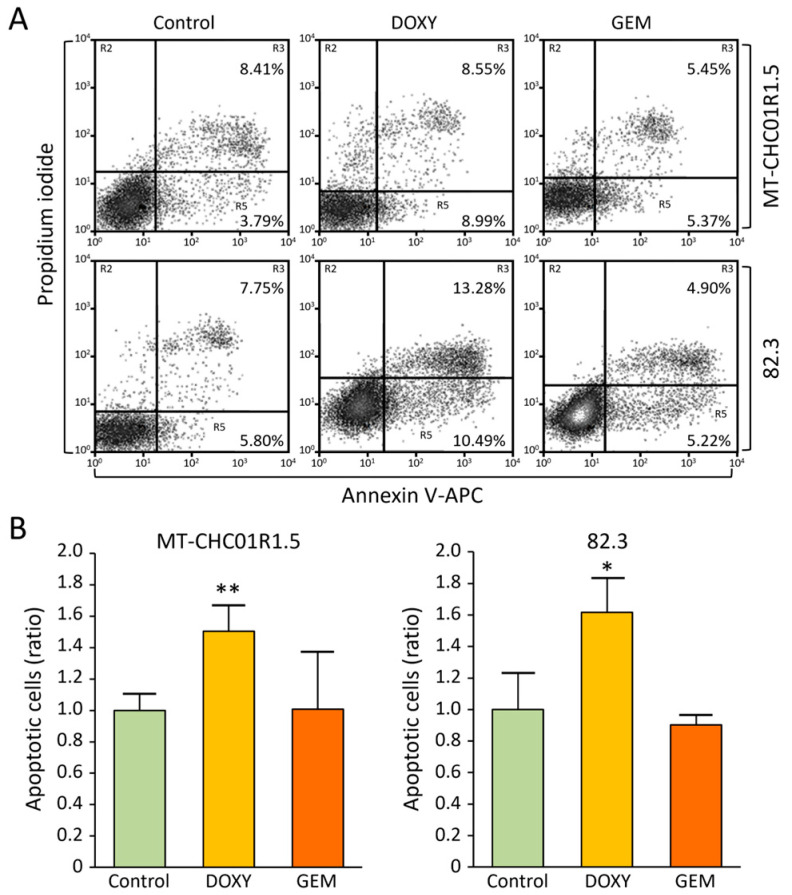
Apoptosis assay. (**A**) The percentages of cells in early apoptosis (Annexin V-positive only) and late apoptosis (Annexin V- and PI-double-positive) are shown. These data are representative of three independent flow cytometry experiments with consistent results. (**B**) The average percentage of Annexin V-positive cells was used to calculate the treatment-to-control ratio. Histograms display the mean ± SD from three independent experiments. Control: no treatment; DOXY: doxycycline; GEM: gemcitabine. * *p* < 0.05 and ** *p* < 0.01 for DOXY vs. Control (one-way ANOVA).

In both cell lines, doxycycline treatment resulted in the emergence of a sub-G1 peak, with values of 2.88 ± 0.87% for MT-CHC01R1.5 and 2.98 ± 0.22% for 82.3 cells (mean ± SD from three analyses), suggesting potential cell death. To further assess apoptosis, we stained the cells with APC-labeled Annexin V (an early apoptosis marker) and PI (a late apoptosis marker), followed by flow cytometry analysis (Figure 2A). The ratio of apoptotic cells was then calculated at different experimental points (Figure 2B). This analysis revealed that doxycycline significantly increased the proportion of apoptotic cells in both cell lines compared to untreated controls. As expected, gemcitabine had no effect on either cell line, consistent with our previous findings [27,28].

### 3.2. Doxycycline Reduces the Stem Cell Subpopulation in Multidrug-Resistant iCCA Cell Lines

To characterize the impact of doxycycline on multidrug-resistant iCCA cells, variations in the stem cell compartment were assessed using the tumor sphere formation assay. In this assay, only cancer cells with stemness properties can form spheroidal aggregates—known as cholangiospheres in the context of iCCA—when maintained in suspension.

**Figure 3:**
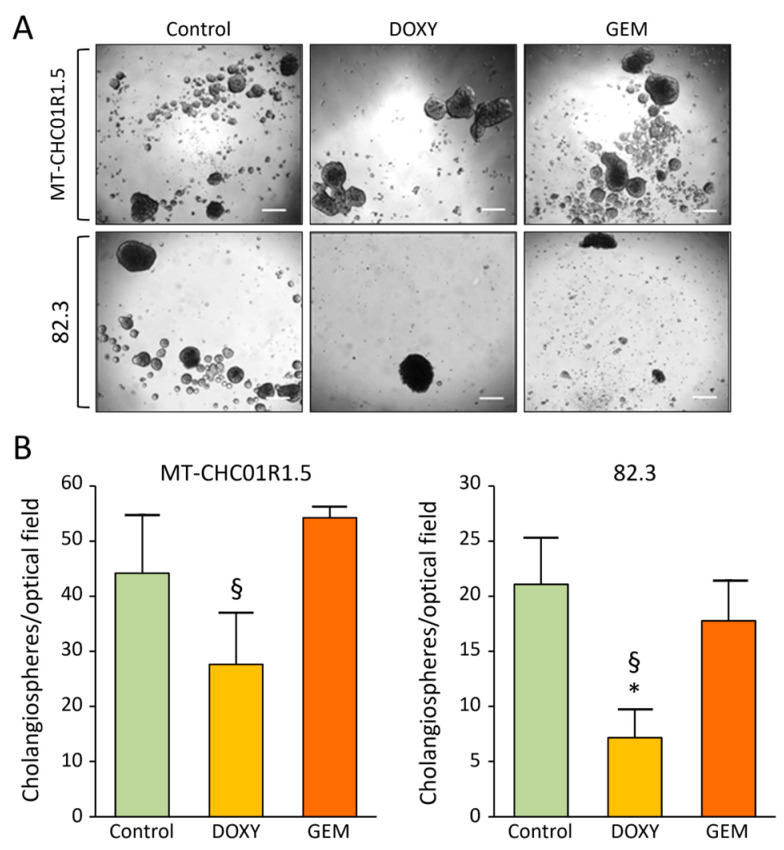
Tumor sphere formation assay. (**A**) Representative images of spheres formed by MT-CHC01R1.5 and 82.3 cells after 7 days of treatment with the indicated drugs. Scale bar, 200 μm. (**B**) Cholangiospheres with a diameter greater than 50 µm were counted across three separate optical fields. Results are presented as the mean ± S from three independent experiments. Control: no treatment; DOXY: doxycycline; GEM: gemcitabine. * *p* < 0.05 for DOXY vs. Control; § *p* < 0.05 for DOXY vs. GEM (one-way ANOVA).

After 7 days of doxycycline treatment, the number of cholangiospheres was reduced in both the MT-CHC01R1.5 and 82.3 cell lines (Figure 3), suggesting a potential impairment of the cancer stem cell subpopulation. As expected, in this assay, gemcitabine had no effect on either cell line.

### 3.3. Doxycycline Restores the Sensitivity to Gemcitabine in Multidrug-Resistant iCCA Xenografts

**Figure 4:**
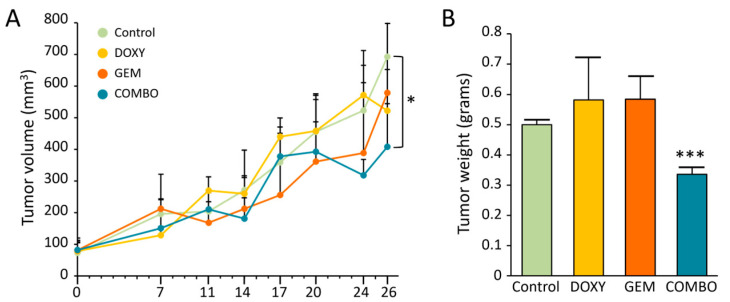
In vivo growth of MT-CHC01R.1.5 xenografted tumor models. Mice were divided into four groups, with treatments administered twice weekly for a total of 26 days. (**A**) Tumor volumes measured with a caliper at the indicated time points. (**B**) Tumor weights measured at the endpoint (day 26). Results are presented as the mean ± SEM for each treatment group. Control: no treatment; DOXY: doxycycline; GEM: gemcitabine; COMBO: doxycycline +gemcitabine. * *p* < 0.05 and *** *p* < 0.001 for COMBO vs. Control (one-way ANOVA).

Having demonstrated that doxycycline affects both viability and stemness potential in multidrug-resistant iCCA cell lines, we investigated whether incorporating this drug into treatment regimens with gemcitabine—the standard first-line therapy for this tumor—could restore therapeutic efficacy in chemoresistant tumors. To evaluate this aspect, we assessed the in vivo activity of doxycycline in NOD/SCID mice xenografted with MT-CHC01R1.5 cells, both as a monotherapy and in combination with gemcitabine. Once the tumors reached a palpable size (~80–100 mm^3^), the mice were randomized into four groups to receive either the drug vehicle, doxycycline (20 mg/kg), gemcitabine (25 mg/kg), or a combination of both, administered intravenously twice weekly until day 26, when the animals were euthanized. At the endpoint, the combination treatment resulted in a significant reduction in both tumor volumes (Figure 4A) and weights (Figure 4B) compared to the vehicle-treated group.

In this experimental setting, neither doxycycline nor gemcitabine alone significantly affected tumor growth in terms of volume or weight, highlighting the necessity of their combination to achieve a significant therapeutic response.

**Figure 5:**
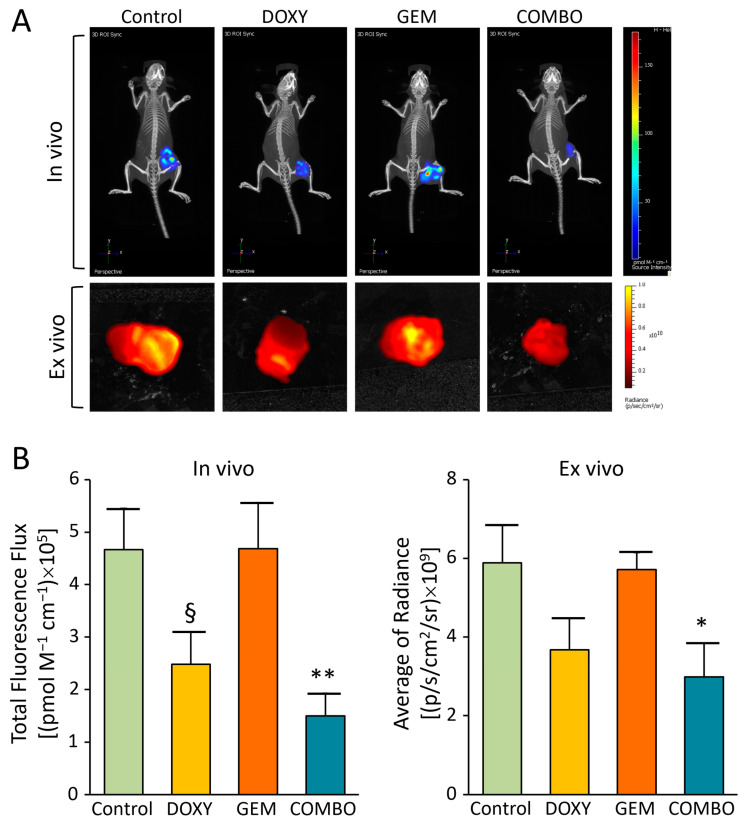
In vivo and ex vivo metabolic activity in xenografted tumor models. (**A**) Representative images showing tumor uptake of 2-DG 750, acquired using an IVIS instrument in live animals (in vivo) and in explanted tumors (ex vivo). (**B**) Cumulative analysis of the total fluorescence flux in tumor masses (in vivo) and the average radiance values (ex vivo). Results are expressed as the mean ± SEM for each treatment group, based on three independent experiments. Control: no treatment; DOXY: doxycycline; GEM: gemcitabine; COMBO: doxycycline + gemcitabine. § *p* < 0.05 for DOXY vs. Control, * *p* < 0.05, ** *p* < 0.01 for COMBO vs. Control (one-way ANOVA).

Finally, we evaluated the in vivo response to doxycycline-based treatments by assessing tumor metabolism through the measurement of glucose uptake in the four arms of treatment described above. At the end of the treatment period, the mice were injected intravenously with a fluorescent glucose derivative, 2-DeoxyGlucose-750, followed by live imaging using an IVIS instrument (Figure 5A). Glucose uptake was also visualized ex vivo in tumors explanted immediately after euthanasia (Figure 5B). The analysis revealed that doxycycline significantly reduces glucose uptake, both as a monotherapy and, more effectively, in combination with gemcitabine. Given that gemcitabine alone is ineffective in our iCCA models, these findings further confirm that combining doxycycline with gemcitabine can overcome drug resistance in this preclinical setting.

## 4. Discussion

This study highlights the potential of doxycycline as a therapeutic agent to overcome MDR in iCCA. We showed that doxycycline significantly reduces cell viability, induces apoptosis, and diminishes the cancer stem cell population in two iCCA cell lines with primary and acquired MDR. Notably, the combination of doxycycline with gemcitabine, the standard first-line chemotherapy for iCCA, restores drug sensitivity thus leading to significant tumor reduction in a xenograft model. These findings suggest that doxycycline could be an effective component of combination therapy aimed at addressing the clinical challenge of chemotherapy resistance in iCCA.

Our results align with previous studies showing the antitumor activity of doxycycline in other cancer types. Cell growth data demonstrate that cell lines were responsive to doxycycline with relatively low IC_50_ values compared to that identified in other tumor models, ranging from 10 to 40 µg/mL in lung [30] and pancreatic [18] cancer, respectively. In our studies, doxycycline did not impact on the cell cycle, but it had a proapoptotic effect on both cell lines. Similarly, in a pancreatic cancer cell line [18], doxycycline has been shown to inhibit proliferation by reducing the expression of anti-apoptotic proteins. Additionally, a recent in vitro study has shown evidence that doxycycline, in combination with doxorubicin, induces apoptosis of prostate cancer cells by increasing BAX expression and decreasing Bcl-2 [10].

We also observed that doxycycline treatment leads to a decrease in the putative cancer stem cell niche. Cancer stem cells play a key role in the development of chemoresistance and, given their characteristic of senescence, they escape chemotherapy treatment thus increasing tumor growth, metastasis, and recurrence [31]. In our MDR iCCA models, doxycycline was able to reduce the formation of cholangiospheres in both cell lines. Our results are in agreement with those obtained in other in vitro cancer models [25,30]. In particular, doxycycline inhibited sphere formation ability and cancer stem cell marker expression in pancreatic cancer cells [13,18]. Additionally, in models of breast cancer, doxycycline reduces both the formation of mammospheres and the CD44+ cancer stem compartment [23]. Recent studies have revealed that CD44 or CD24 silencing decreased invasive capacity, migration, and cell adhesion in CCA cell lines [32,33], suggesting that targeting this cell subpopulation may represent a potential therapeutic strategy for CCA.

The in vivo findings reported here support the translational potential of doxycycline in overcoming MDR. The significant reduction in tumor volume and weight with the combination therapy, compared to either agent alone, highlights the synergistic effect of doxycycline and gemcitabine. This synergy is particularly noteworthy given that gemcitabine alone was ineffective, indicating that doxycycline may sensitize resistant cancer cells to conventional chemotherapy. Interestingly, the observed reduction in glucose uptake in doxycycline-treated tumors suggests that metabolic reprogramming may be a key mechanism through which doxycycline exerts its antitumor effects. Cancer cells, particularly those with stem cell-like properties, often depend on altered metabolic pathways for growth and survival [34]. By disrupting these pathways, doxycycline may weaken the tumor’s metabolic adaptability, rendering it more susceptible to chemotherapeutic agents. Consistent with these observations, two studies on pancreatic [35] and lung [36] cancer cell lines suggest that doxycycline may enhance gemcitabine efficacy by modulating mitochondrial functionality, specifically by increasing susceptibility to apoptosis, reducing mitochondrial protein synthesis, and decreasing ATP production, thereby lowering overall energy metabolism.

To date, there are no preclinical or clinical studies specifically investigating the in vivo antitumor activity of doxycycline in CCA. Although our xenograft model provides valuable insights, clinical validation will be essential to confirm whether these preclinical results can be translated into effective therapies for patients. Future studies should explore the optimal dosing regimen and potential side effects of doxycycline in combination with gemcitabine, especially given doxycycline’s long history of use as an antibiotic and its well-established safety profile.

## 5. Conclusions

This work provides the first scientific basis for considering doxycycline as a potential antitumor agent in iCCA patients with intrinsic or acquired resistance to standard therapies. By targeting both the bulk tumor and the cancer stem cell compartment, doxycycline may enhance the efficacy of existing chemotherapies and improve outcomes for patients with this aggressive and hard-to-treat cancer.

## Data Availability

The raw data supporting the conclusions of this article will be made available by the authors on request.
